# Supplementary material for: Upc2-mediated mechanisms of azole resistance in Candida auris
Source: Microbiol Spectr. 2024 Jan 11;12(2):e03526-23. doi: 10.1128/spectrum.03526-23 (PMC10845950; doi:10.1128/spectrum.03526-23)
Supplement: Supplemental material — Tables S1 and S2; Figures S1 to S8. [file spectrum.03526-23-s0001.pdf]

Upc2-mediated mechanisms of azole resistance in *Candida auris*

Jizhou LI[1,2†], Lola AUBRY[1†], Danielle BRANDALISE[1], Alix T. COSTE[1], Dominique SANGLARD[1], Frederic LAMOTH[1,2]

[1] Institute of Microbiology, Lausanne University Hospital and University of Lausanne, Lausanne, Switzerland

[2] Infectious Diseases Service, Department of Medicine, Lausanne University Hospital and University of Lausanne, Lausanne, Switzerland

[†] These authors equally contributed to the work.

Supplementary Material

Supplementary Table 1: Plasmids used in this study

Name	Description	Reference
pJK795	*NatR* selection marker for nourseothricin	(26)
pYM70	*HygR* selection marker for hygromycin	(27)
Clp-p*ACT1*-3xFLAG-MNase-SV40-*CYC-SAT1*	*CauNi* and SAT1 selection marker for hygromycin	(28)
pjli6	P_{ADH1}, *CauNi* and *SAT1* selection marker for nourseothricin	This study
pjli7	P_{ADH1}, *MRR1-3XHA* Tag, *CauNi* and *SAT1* selection marker for nourseothricin	This study
pjli8	P_{ADH1}, *MRR1-3XHA* Tag, T_{ACT1}, *CauNi* and *SAT1* selection marker for nourseothricin	This study
pjli11	P_{ADH1}, *TAC1b-3XHA* Tag, T_{ACT1}, *CauNi* and *SAT1* selection marker for nourseothricin	This study
pjli12	P_{ADH1}, *UPC2-3XHA* Tag, T_{ACT1}, *CauNi* and *SAT1* selection marker for nourseothricin	This study

Supplementary Table 2: Primers and RNAs guides used in this study

Primer	Aim	Sequence (5' → 3')
ADH1p_PF_KpnI	*ADH1* promotor amplification containing KpnI restriction site to clone pjli6	ACACTGGTACCCGAGATAGATCGAAATACGCTC
ADH1p_PR_KasI_NheI	*ADH1* promotor amplification containing NheI restriction site to clone pjli6	ACACTGCTAGCGGCGCCGATTTCGTGAAGATTGATTGATGATG
MRR1_PF_KasI	*MRR1* amplification containing KasI restriction site to clone pjli7	ACACTGGCGCCATGGTATCTTCGAAAGATC TGGC
MRR1_PR_BsrGI_Tag_CS_NruI_NheI	*MRR1* amplification containing BsrGI restriction site, the 3xHA Tag sequence, codon stop of *MRR1*, and NruI, NheI restriction sites to clone pjli7	ACACTGCTAGCTCGCGATTAAGCGTAATCCGGAACATCGTATGGGTAAGCGTAATCCGGAACATCGTATGGGTAAGCGTAATCCGGAACATCGTATGGGTATGTACACACATCAAGCATCTCTTCGAATG
ACT1t_PF_NheI	*ACT1* terminator amplification containing NheI restriction site to clone pjli8	ACACTGCTAGCTCTCTCAAAGATGGTTAGTATTCTTGC
ACT1t_PR_NheI	*ACT1* terminator amplification containing NheI restriction site to clone pjli8	ACACTGCTAGCGAGTGACTGCTGGGTAGTAG
TAC1b_PF_KasI	*TAC1b* amplification containing KasI restriction site to clone pjli11	ACA CTG GCG CCA TGA GTG CAG TGG TGA AGG G

TAC1b_PR_BsrGI	*TAC1b* amplification containing BsrGI restriction site to clone pjli11	ACACTTGTACAAAGCCCATTATCGAAGAAG AAATTAG
UPC2_PF_KasI	*UPC2* amplification containing KasI restriction site to clone pjli12	ACACTGGCGCCATGTCTATGAAAGAAGAGC AACAGC
UPC2_PR_BsrGI	*UPC2* amplification containing BsrGI restriction site to clone pjli12	ACACTTGTACAAATATCGTTAGACCCAATGAACCC
pjli8_ADH1_PF	pjli8, pjli11 and pjli12 sequencing / *TAC1b*, *MRR1*, *UPC2* hyperactivation verification	AGCAACACCGGTGGAATTTCC
MRR1_PrimerF2	pjli8 sequencing	GCTAAGCAGATGTTCGATGCG
MRR1_PrimerF3	pjli8 sequencing	CTCATCCATTTCTGGGAAGCC
MRR1_PrimerF4	pjli8 sequencing	GAATAAGCATACTTTGTCCTTGGG
MRR1_PrimerF5	pjli8 sequencing	TACGTCTTTATCACACTTATGCTCC
MRR1_PrimerF6	pjli8 sequencing / *MRR1* deletion verification	ATCTTTCACTCAAGACATTTTTGATCTC
MRR1_PrimerF7	pjli8 sequencing	AAGTCAAGGAGTTGGTAACCATTTG

TAC1_PrimerF2	pjli11 sequencing	AGCTGAGTCTCCAGGAAACTG
TAC1_PrimerF3	pjli11 sequencing	GAAGCAGTACAAGTTTTTGAACTCG
TAC1_PrimerF4	pjli11 sequencing	GCTGGTAAACATCGTCGACG
TAC1_PrimerF5	pjli11 sequencing	CTTCGCCATGAACTGGATAATTAC
TAC1_PrimerF6	pjli11 sequencing	AAGAATGACCCTACACTTTCCAAC
UPC2_PF2	pjli12 sequencing	CCGCCACCTAACCTATCAAAC
UPC2_PF3	pjli12 sequencing / *UPC2* deletion verification	GCCAGGTTTCCAACACGATTAC
UPC2_PF4	pjli12 sequencing	GCTGAAGTCATTTGCTGATCTCG
CauNi_verif_PF	*TAC1b*, *MRR1*, *UPC2* hyperactivation verification	TGGCCTCTTGATAAGGGCTG
sat1_verif_PR	*TAC1b*, *MRR1*, *UPC2* hyperactivation verification	GAGACTGTGCGCGACTCC
pjli8_ACT1t_PR	*TAC1b*, *MRR1*, *UPC2* hyperactivation verification	GTACGCTAAAGGGTCATGAGC
UPC2_del_PF1	Construction of *UPC2* deletion cassette	CTATCCGTGATAGCTAAGTCGG

UPC2_del_PR1	Construction of *UPC2* deletion cassette	GTATTCTGGGCCTCCATGTCCTCGGGAGAGTTGAATCCTC
UPC2_del_PF2	Construction of *UPC2* deletion cassette	GAGGATTCAACTCTCCCGAGGACATGGAGGCCCAGAATAC
UPC2_del_PR2	Construction of *UPC2* deletion cassette	CCTTCACTACTAACTCTTCACACTCAGTATAGCGACCAGCATTCAC
UPC2_del_PF3	Construction of *UPC2* deletion cassette	GTGAATGCTGGTCGCTATACTGAGTGTGAA GAGTTAGTAGTGAAGG
UPC2_del_PR3	Construction of *UPC2* deletion cassette	AGAGGGGTTGCAAAGGAGAG
UPC2_del_PF4	Construction of *UPC2* deletion cassette	CATGCAGGTGTAGGTTACGAAAG
UPC2_del_PR4	Construction of *UPC2* deletion cassette	TCGTGGTGGAGATTCTCATGG
UPC2_del_PR1(hygro)	Construction of *UPC2* deletion cassette	TATCGAACAGCAAGCACTATACCTCGGGAG AGTTGAATCCTC
UPC2_del_PF2(hygro)	Construction of *UPC2* deletion cassette	GAGGATTCAACTCTCCCGAGGTATAGTGCT TGCTGTTCGATA
UPC2_del_PR2(hygro)	Construction of *UPC2* deletion cassette	CTTCACTACTAACTCTTCACACTATTTTATGATGGAATGAATGGGATG
UPC2_del_PF3(hygro)	Construction of *UPC2* deletion cassette	CATCCCATTCATTCCATCATAAAATAGTGTGAAGAGTTAGTAGTGAAG
NAT1_743_F	*UPC2* deletion verification	AGTTCTTCGTTTCACTGAGTATACG
UPC2_del_verif_PR	*UPC2* deletion verification	CGAGGTAGTGGAAGGAGAAG

F444Lverif_PF	*UPC2*, *MRR1*, *MDR1* deletion verification	CCACCCAAGGCATTTCTATATC
MRR1del_PF1	*MRR1* deletion	CGCTCCACTCTTAGAAAAATGGTC
MRR1del_PR1	*MRR1* deletion	TATCGAACAGCAAGCACTATACGAGAAGTGTTAATTGCCGCT
MRR1del_PF2	*MRR1* deletion	AGCGGCAATTAACACTTCTCGTATAGTGCTTGCTGTTCGATA
MRR1del_PR2	*MRR1* deletion	CGCATGTATATTTACGTAGTATGATTTTATGATGGAATGAATGGGATG
MRR1del_PF3	*MRR1* deletion	CATCCCATTCATTCCATCATAAAATCATACTACGTAAATATACATGCG
MRR1del_PR3	*MRR1* deletion	TTGGTCATAAACTCAATATCCCTCC
MRR1del_PF4	*MRR1* deletion	GCATCTTCCATGACTAGACGC
MRR1del_PR4	*MRR1* deletion	CATAGTCAGGTAGAGAGCCTTC
MRR1del_Verif_PR2	*MRR1* deletion verification	CACCAGATTGGAGTTCGGTAAG
MRR1_PrimerR2	*MRR1* deletion verification	TTGTGGGTGTTATCGGTGCC
MDR1del_PF1	*MDR1* deletion	AAATAGGCAGGCGGGCAACG
MDR1del_PR1	*MDR1* deletion	TATCGAACAGCAAGCACTATACGTGGAGATTGAAGATGCGTTG
MDR1del_PF2	*MDR1* deletion	CAACGCATCTTCAATCTCCACGTATAGTGCTTGCTGTTCGATA
MDR1del_PR2	*MDR1* deletion	GATCAGGGCTACATCGTCTTATTTTATGATGGAATGAATGGGATG
MDR1del_PF3	*MDR1* deletion	CATCCCATTCATTCCATCATAAAATAAGACGATGTAGCCCTGATC
MDR1del_PR3	*MDR1* deletion	GGAGGTTTCATCCGCAAAAGC
MDR1del_PF4	*MDR1* deletion	CCGGTGGTGGTTCACGTAAG
MDR1del_PR4	*MDR1* deletion	CGGCTGCGAAAAGAGGTTCC
MDR1del_verif_PR	*MDR1* deletion verification	CGCTGCCAAAGTGCTAATATCC
MDR1_verif_PF	*MDR1* deletion verification	GTCTGTCAAGGCTGACTACC
ACT1_F	*ACT1* RT_PCR	GAAGGAGATCACTGCTTTAGCC
ACT1_R	*ACT1* RT_PCR	GAGCCACCAATCCACACAG
UPC2_PF(sybrgreen)	*UPC2* RT_PCR	AGGGTGTGTCACAGGTGTTG

UPC2_PR(sybrgreen)	*UPC2* RT_PCR	CGCCTCCCAAGAAATCAAGC
ERG11_PF(sybrgreen)	*ERG11* RT_PCR	GTTTGCCTACGTGCAATTGG
ERG11_PR(sybrgreen)	*ERG11* RT_PCR	GTAGTCGACTGGTGGAAGCG
TAC1b_PF(sybrgreen)	*TAC1b* RT_PCR	AGAAGTGAACGCTCCTTCCG
TAC1b_PR(sybrgreen)	*TAC1b* RT_PCR	GGCGTGAATTTCCTGTCGTC
CDR1_F	*CDR1* RT_PCR	GAAATCTTGCACTTCCAGCCC
CDR1_R	*CDR1* RT_PCR	CATCAAGCAAGTAGCCACCG
MRR1_PF(sybrgreen)	*MRR1* RT_PCR	TAGAGCCACTACCATCCGAC
MRR1_PR(sybrgreen)	*MRR1* RT_PCR	AGTTTGTGCCCTGCCTGATG
MDR1_F	*MDR1* RT_PCR	GAAGTATGATGGCGGGTG
MDR1_R	*MDR1* deletion verification/*MDR1* RT_PCR	CCCAAGAGAGACGAGCCC
CauNi_sg5’	Guide RNA for *TAC1b*, *MRR1*, *UPC2* hyperactivation	CCCGGAGAUACACGGCGCCG
CauNi_sg3’	Guide RNA for *TAC1b*, *MRR1*, *UPC2* hyperactivation	GCUGCAAAAUAAGGCCAGAG
UPC2_del_sg5’	Guide RNA for *UPC2* deletion	AAUCUUCUGAGAAAAAGAGA
UPC2_del_sg3’	Guide RNA for *UPC2* deletion	AGCAACUUGGACAUCAUGCA
MRR1del_Guide_RNA_5'	Guide RNA for *MRR1* deletion	UUGACGCGAGAGAAUUUGGA
MRR1del_Guide_RNA_3'	Guide RNA for *MRR1* deletion	ACGCCUGGAGAAUCACUAGA
MDR1del_sg5'	Guide RNA for *MDR1* deletion	CAAAGUGUUCACAUACCCCG
MDR1del_sg3'	Guide RNA for *MDR1* deletion	CCAGUAUUAUUCUACCUCAA

Supplementary Figure 1

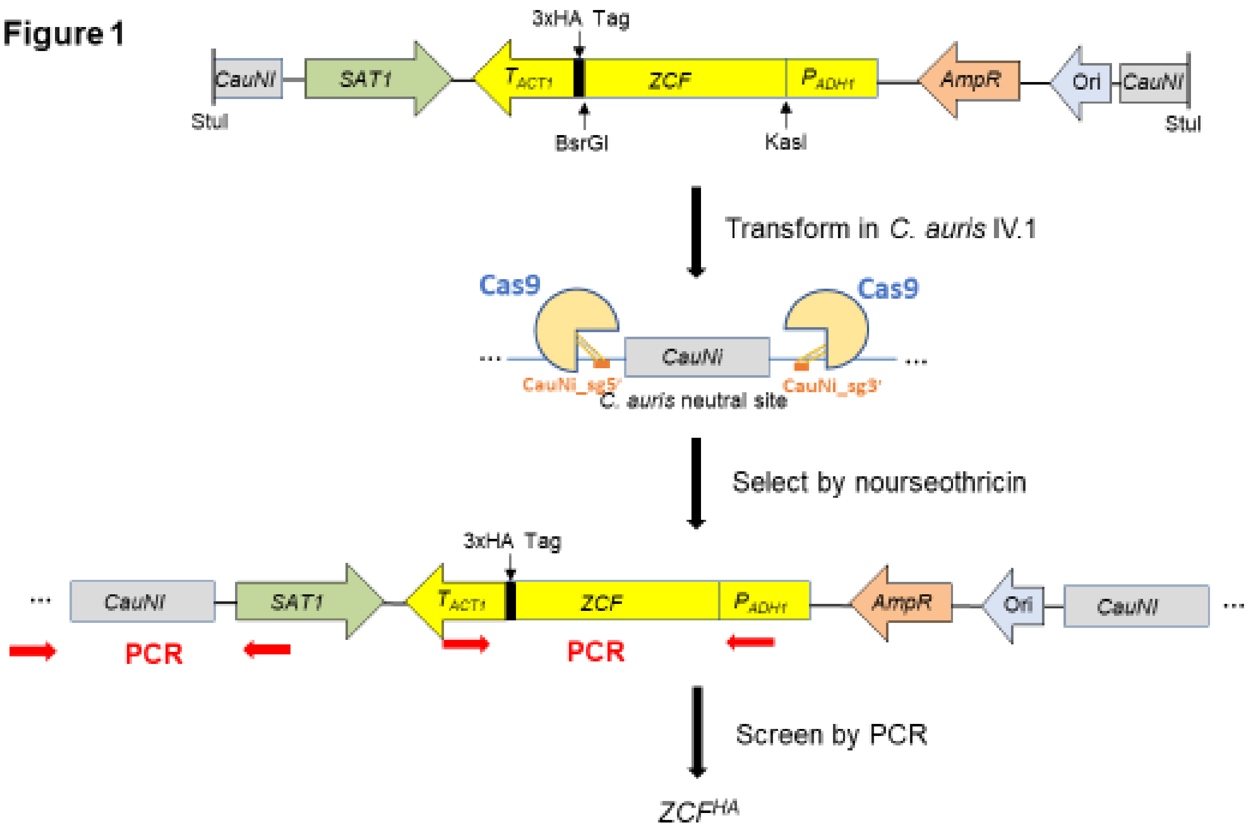

Schematic view of the zinc cluster transcription factor (ZCF) hyperactivation system in *C. auris*. The plasmid containing the promotor P_{ADH1}, the nucleotide sequence of ZCF fused by 3xHa Tag, the terminator of T_{ACT1}, the SAT1 cassette (nourseothricin resistance) and the *C. auris* neutral site *CauNI* was linearized by StuI. The restriction sites KasI and BsrGI were used to insert the ZCF nucleotide sequences. The linearized plasmid was transformed via electroporation in the wild-type IV.1 strain with CRISPR-Cas9 method, by targeting the upstream and the downstream regions of *CauNI* with the nucleotide-specific guide RNAs CauNi_sg5' and CauNi_sg3'.

Supplementary Figure 2A

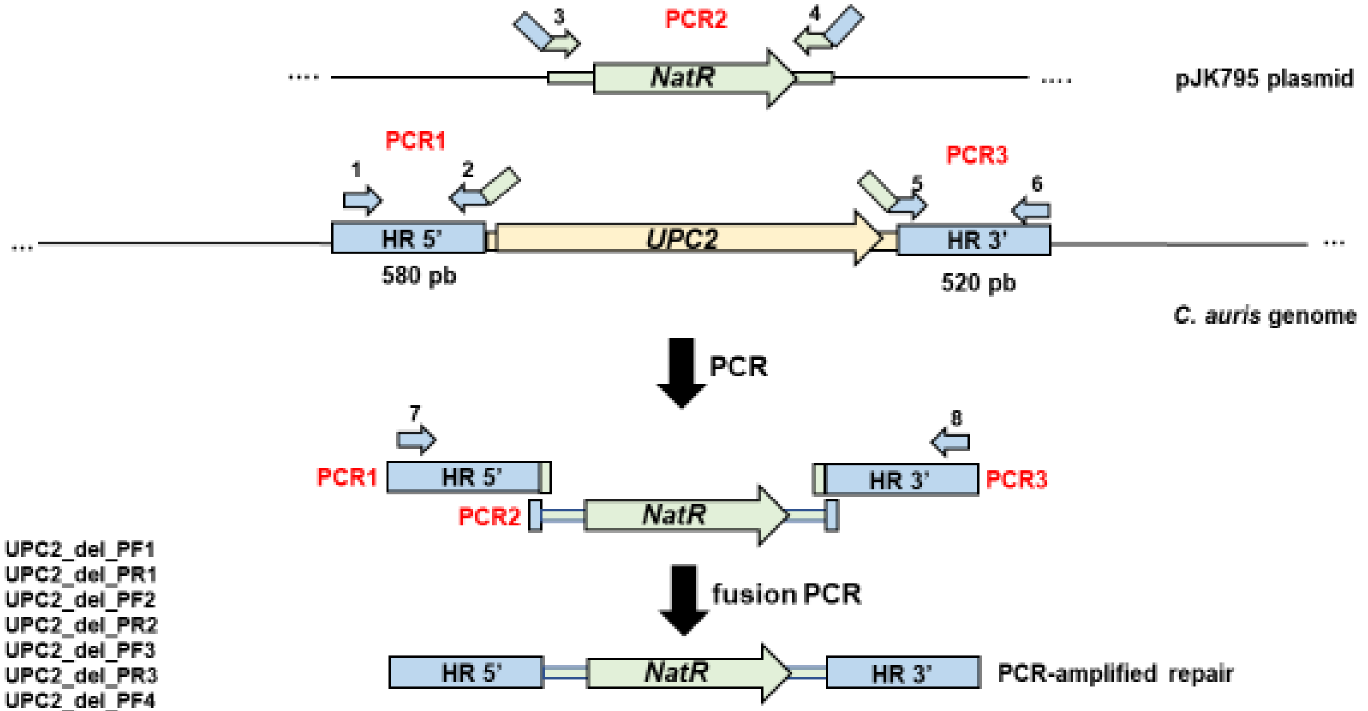

Primer 1: UPC2_del_PF1
Primer 2: UPC2_del_PR1
Primer 3: UPC2_del_PF2
Primer 4: UPC2_del_PR2
Primer 5: UPC2_del_PF3
Primer 6: UPC2_del_PR3
Primer 7: UPC2_del_PF4
Primer 8: UPC2_del_PR4

Schematic view of the fusion PCRs for *UPC2* deletion in the IV.1 strain to generate the *upc2*Δ strain. The fragment contains the *NatR* cassette (nourseothricin resistance) and 500 pb flanking sequences (HR 5' and HR 3') of *UPC2*. The fusion PCRs were carried out with overlapping primers as indicated. Primer 2 is reverse complemented to primer 3, and primer 4 is reverse complemented to primer 5.

Supplementary Figure 2B

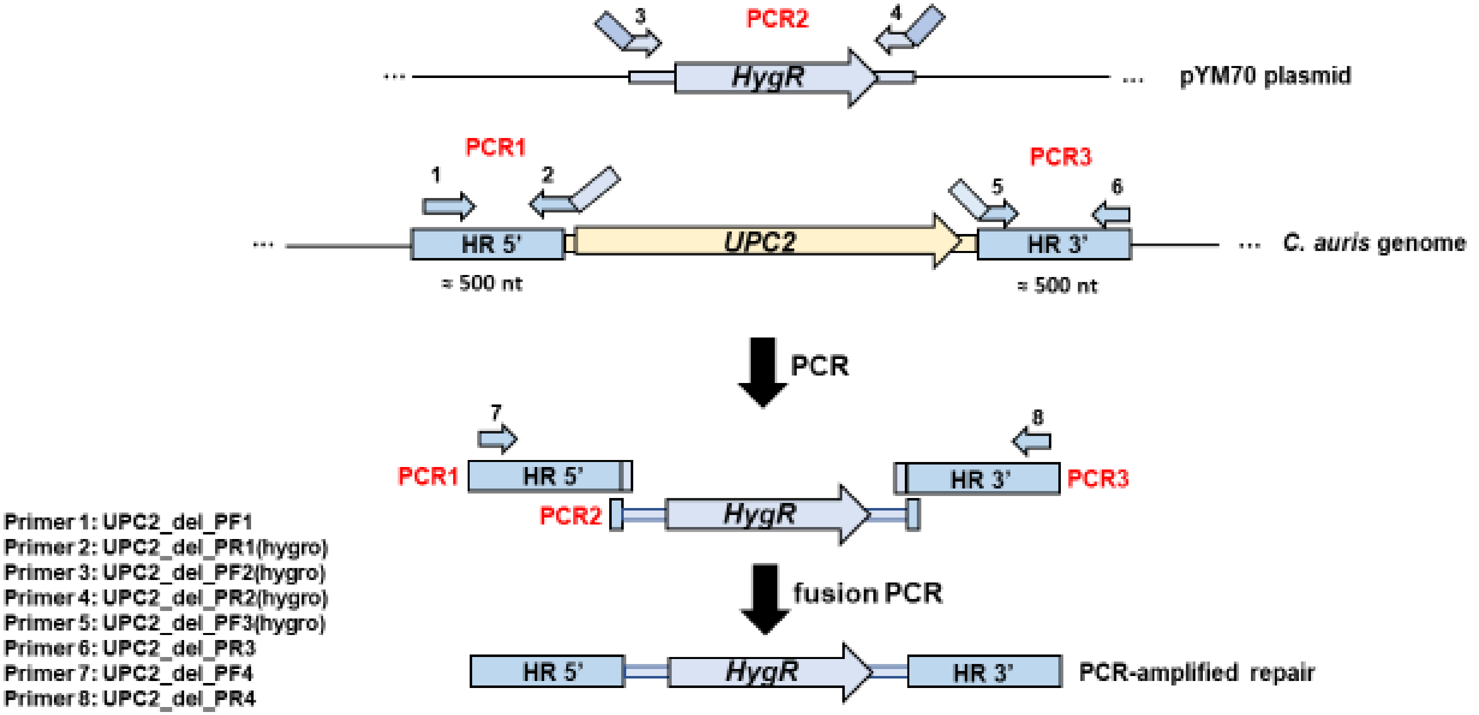

Primer 1: UPC2_del_PF1
Primer 2: UPC2_del_PR1(hygro)
Primer 3: UPC2_del_PF2(hygro)
Primer 4: UPC2_del_PR2(hygro)
Primer 5: UPC2_del_PF3(hygro)
Primer 6: UPC2_del_PR3
Primer 7: UPC2_del_PF4
Primer 8: UPC2_del_PR4

Schematic view of the fusion PCRs for *UPC2* deletion in the *TAC1b^{HA}* and *MRR1^{HA}* strains to generate the *TAC1b^{HA}/upc2Δ* and *MRR1^{HA}/upc2Δ* strains. The fragment contains the *HygR* cassette (hygromycin resistance) and 500 pb flanking sequences (HR 5' and HR 3') of *UPC2*. The fusion PCRs were carried out with overlapping primers as indicated. Primer 2 is reverse complemented to primer 3, and primer 4 is reverse complemented to primer 5.

Supplementary Figure 3

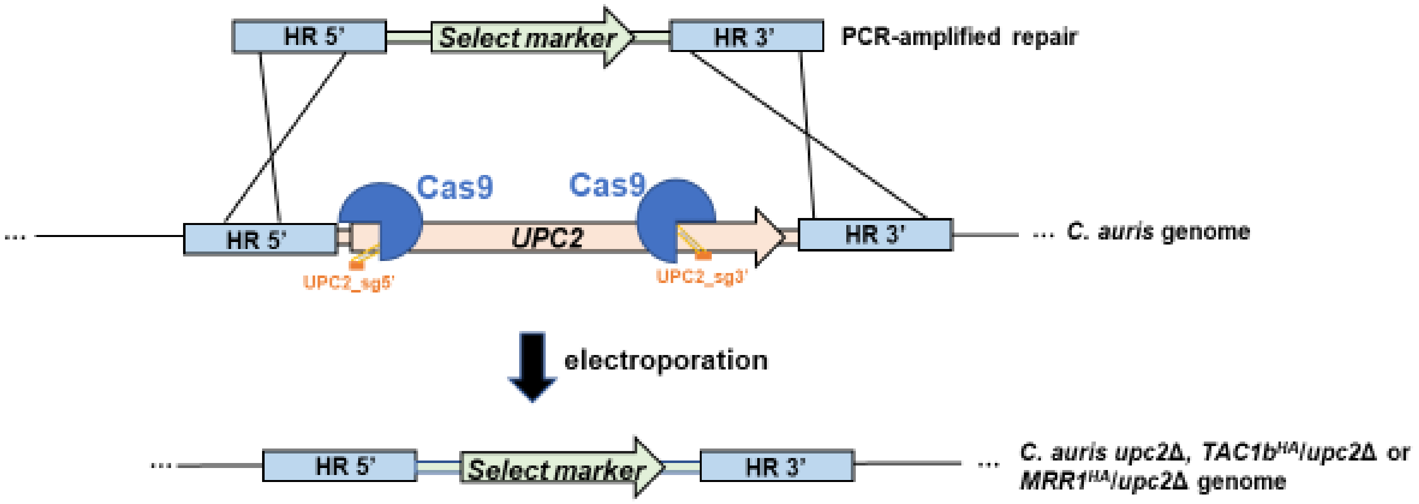

Principle for CRISPR-Cas9-mediated deletion of *UPC2* in the IV.1, *TAC1b*^{HA} and *MRR1*^{HA} strains. Two guide-RNAs were used: UPC2_sg5' and UPC2_sg3'. The selection markers *NatR* (nourseothricin resistance) and *HygR* (hygromycin resistance) were used for *UPC2* deletion in strain IV.1 and in strains *TAC1b*^{HA} and *MRR1*^{HA}, respectively.

Supplementary Figure 4A

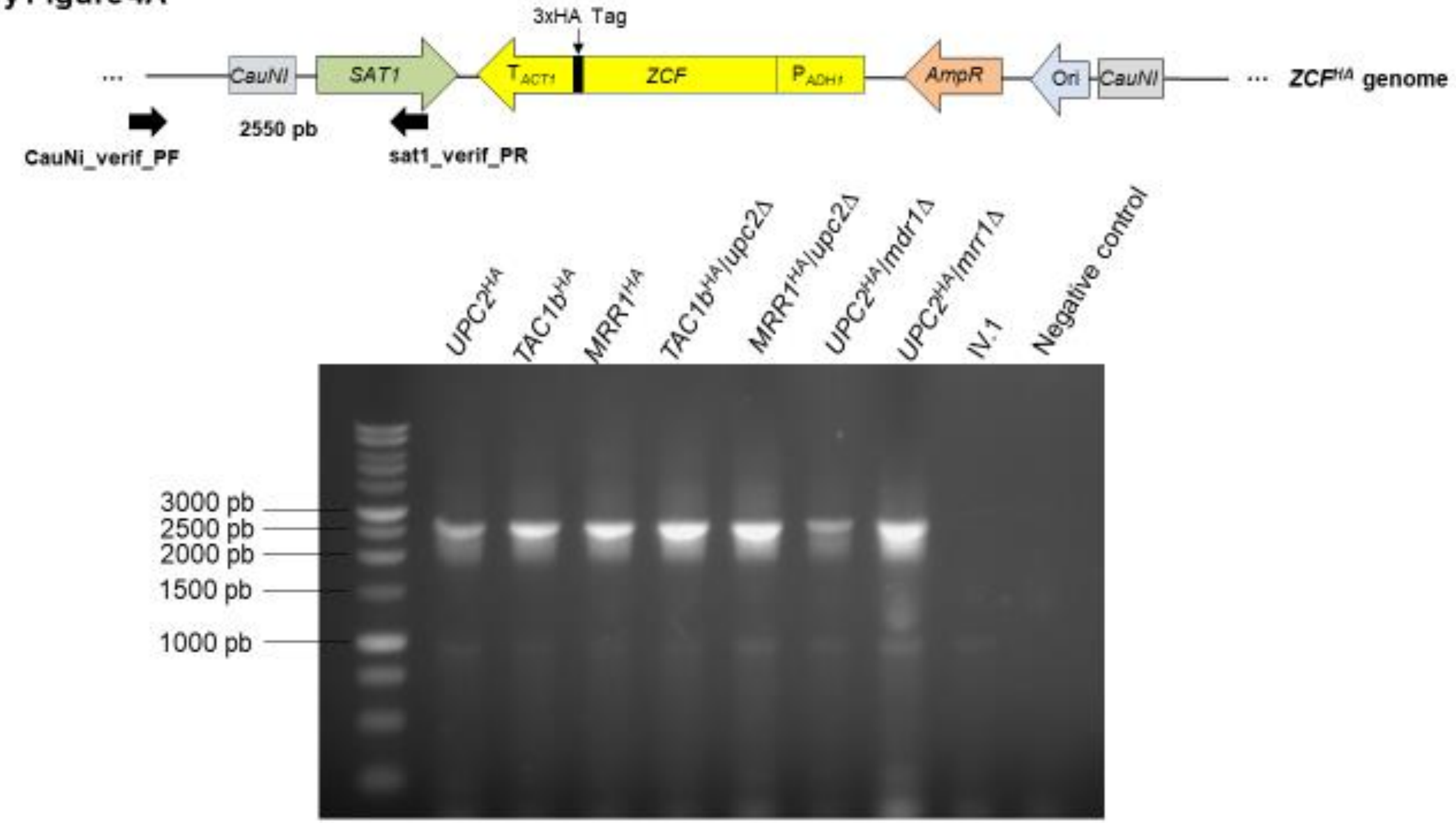

Verification of correct integration of the transformation cassette for zinc cluster transcription factor hyperactivation in the *UPC2^{HA}*, *TAC1b^{HA}*, *MRR1^{HA}*, *TAC1b^{HA}/upc2*Δ, *MRR1^{HA}/upc2*Δ, *UPC2^{HA}/mdr1*Δ and *UPC2^{HA}/mrr1*Δ strains. An approximately 2.5 kb fragment was amplified with one primer in *SAT1* and another primer in the upstream region of the *C. auris* neutral site *CauNI*. As shown on the electrophoresis gel, the PCR product was present in the mutant strains and absent in the IV.1 strain.

Supplementary Figure 4B

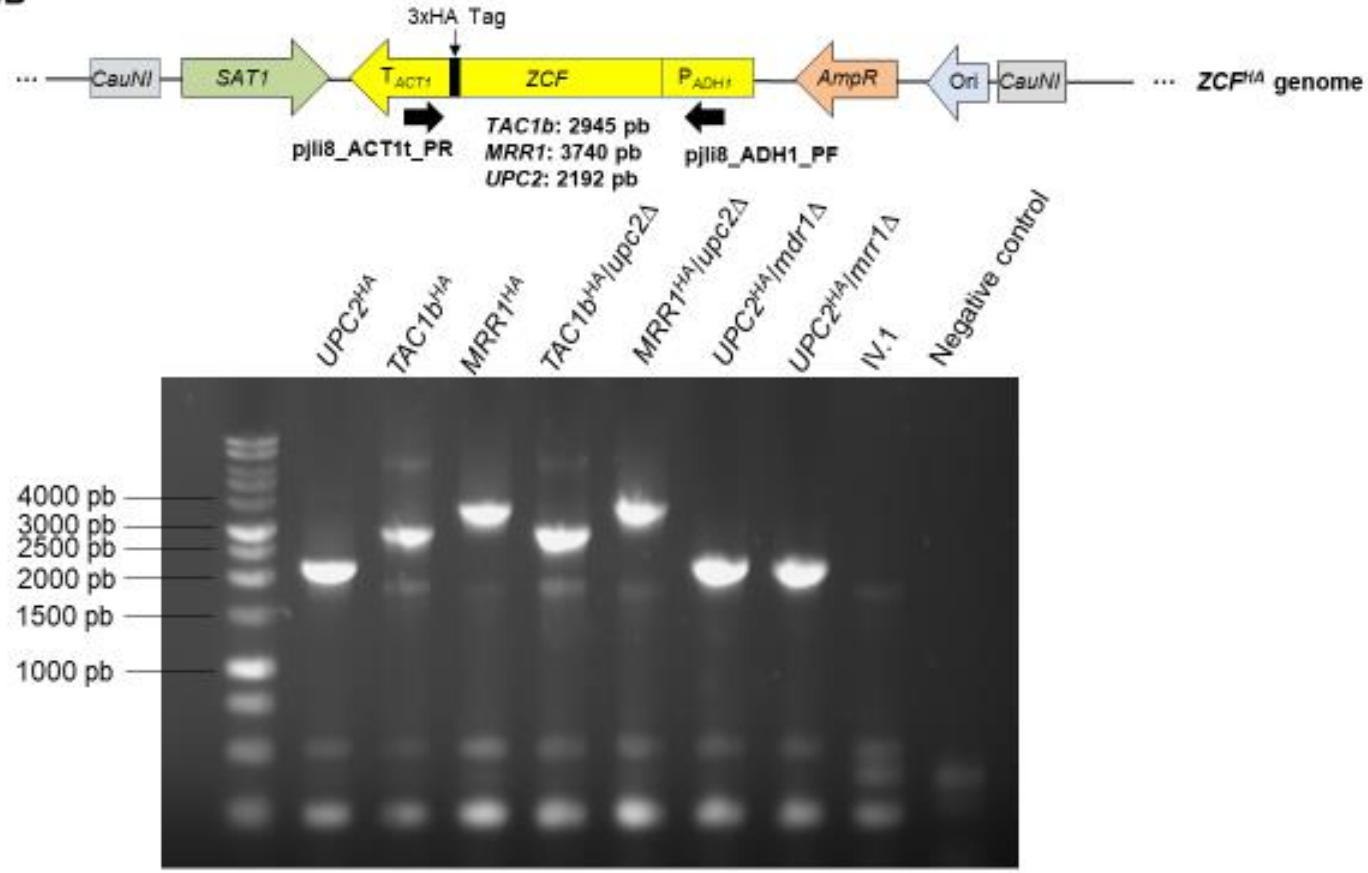

Verification of correct integration of the transformation cassette for zinc cluster transcription factor hyperactivation in the *UPC2*^{HA}, *TAC1b*^{HA}, *MRR1*^{HA}, *TAC1b*^{HA}*/upc2*Δ, *MRR1*^{HA}*/upc2*Δ, *UPC2*^{HA}*/mdr1*Δ and *UPC2*^{HA}*/mrr1*Δ strains. A fragment was amplified with one primer in the promoter P_{ADH1} and another primer in the terminator T_{ACT1}. As shown on the electrophoresis gel, PCR products of different sizes (depending on the length of the target genes) were obtained in all the mutant strains.

Supplementary Figure 5

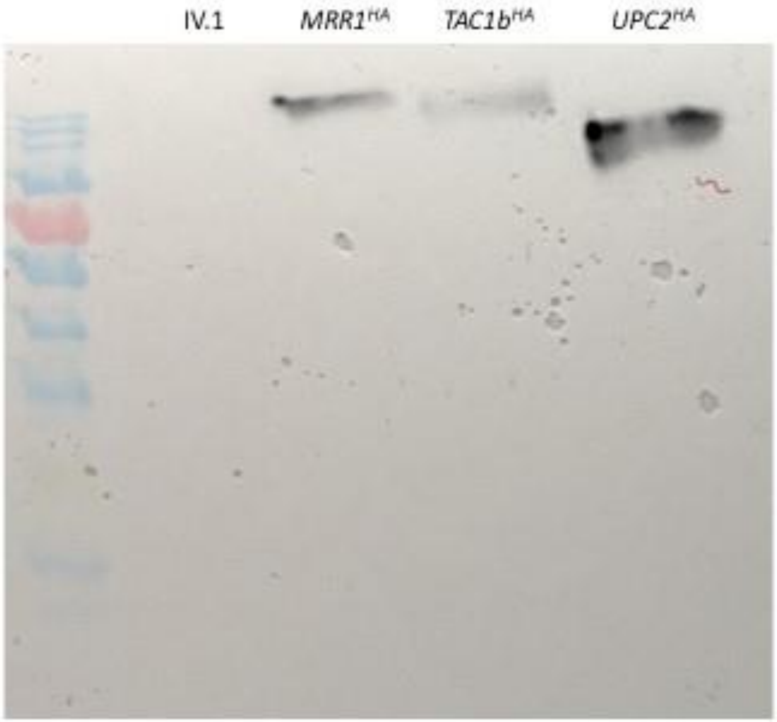

Verification of the HA tagging of Mrr1p, Tac1bp and Upc2p in the *MRR1^{HA}*, *TAC1b^{HA}* and *UPC2^{HA}* strains, respectively. Western blot analysis using a HA tag monoclonal antibody showed the presence of detectable bands at the expected size: 129 kDa, 99 kDa and 67 kDa, in the *MRR1^{HA}*, *TAC1b^{HA}* and *UPC2^{HA}* strains, respectively.

Supplementary Figure 6A

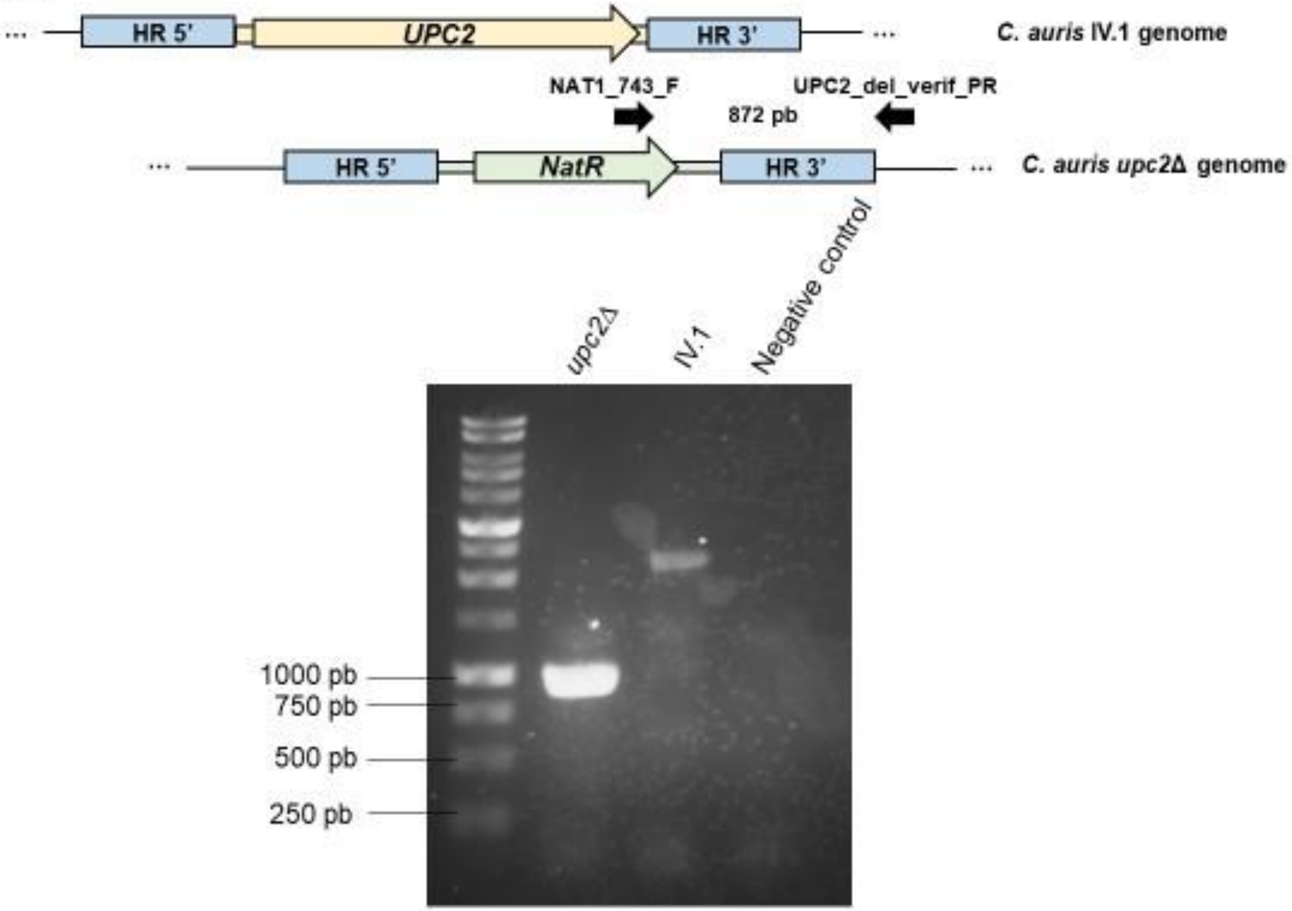

Verification of the deletion of *UPC2* in the *upc2*Δ strain. An approximately 900 pb fragment was amplified with one primer in *NatR* and another primer in the downstream region of HR 3'. As shown on the electrophoresis gel, the PCR product was present in the mutant strain and absent in the IV.1 strain.

Supplementary Figure 6B

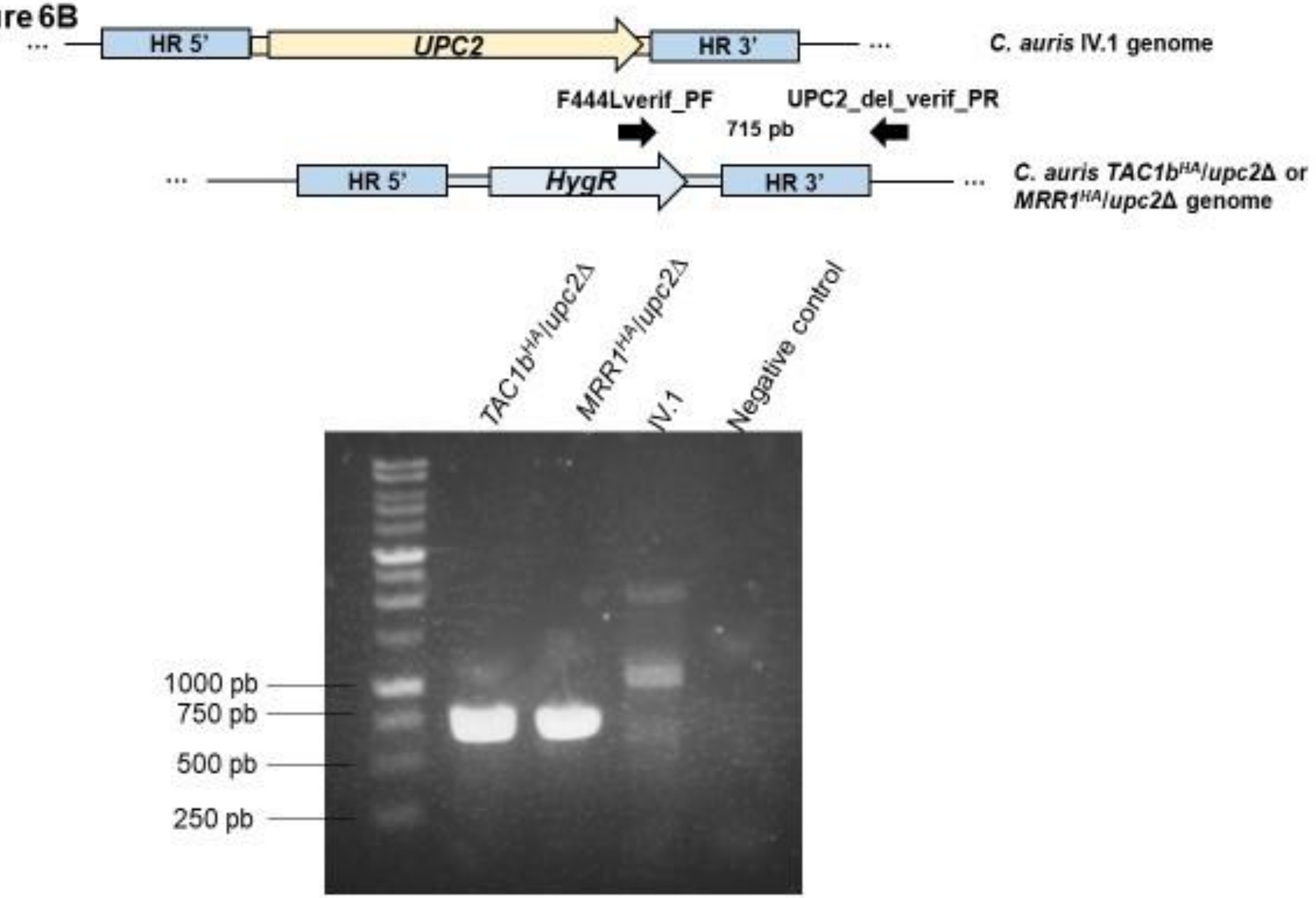

Verification of the deletion of *UPC2* in the *TAC1b^{HA}/upc2Δ* and *MRR1^{HA}/upc2Δ* strains. An approximately 700 pb fragment was amplified with one primer in *HygR* and another primer in the downstream region of HR 3'. As shown on the electrophoresis gel, the PCR product was present in the mutant strains and absent in the IV.1 strain.

Supplementary Figure 6C

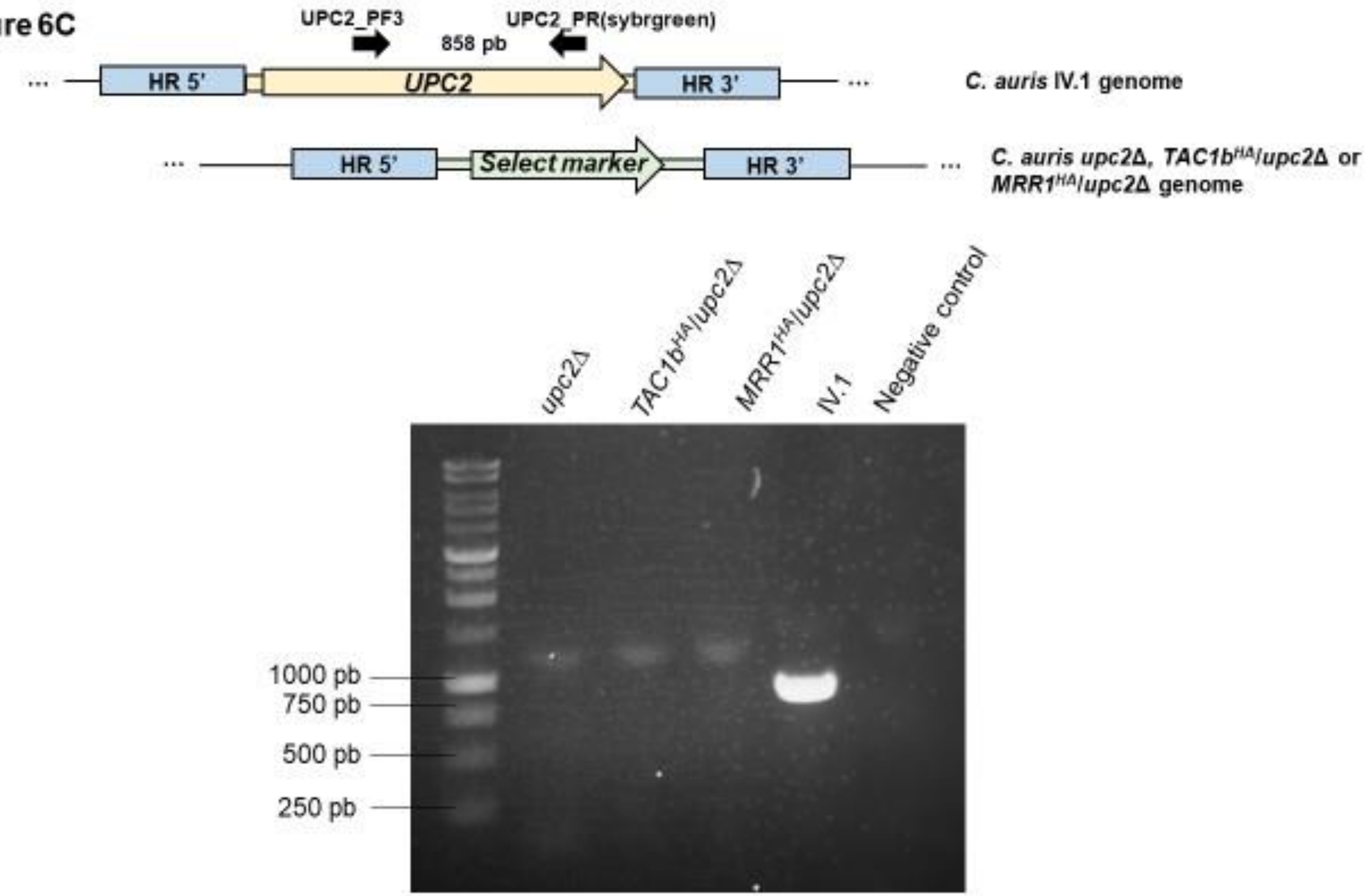

Verification of the deletion of *UPC2* in the *upc2Δ*, *TAC1b^HA/upc2Δ* and *MRR1^HA/upc2Δ* strains. An approximately 800 pb fragment was amplified with two primers located within *UPC2*. As shown on the electrophoresis gel, the PCR product was present in the IV.1 strain and absent in the mutant strains.

Supplementary Figure 7A

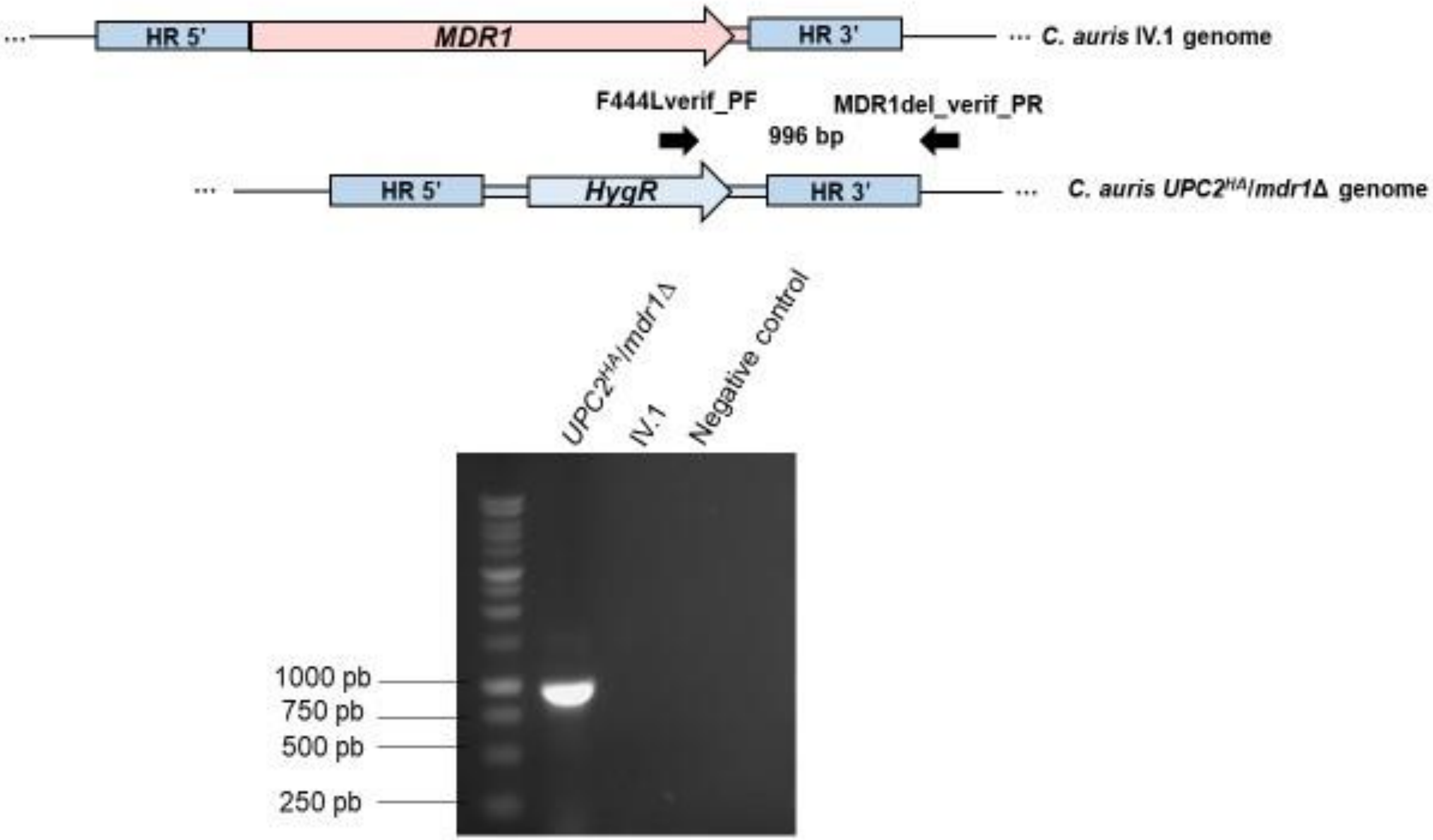

Verification of the deletion of *MDR1* in the *UPC2^{HA}/mdr1Δ* strain. An approximately 1 kb fragment was amplified with one primer in *HygR* and another primer in the downstream region of HR 3'. As shown on the electrophoresis gel, the PCR product was present in the mutant strain and absent in the IV.1 strain.

Supplementary Figure 7B

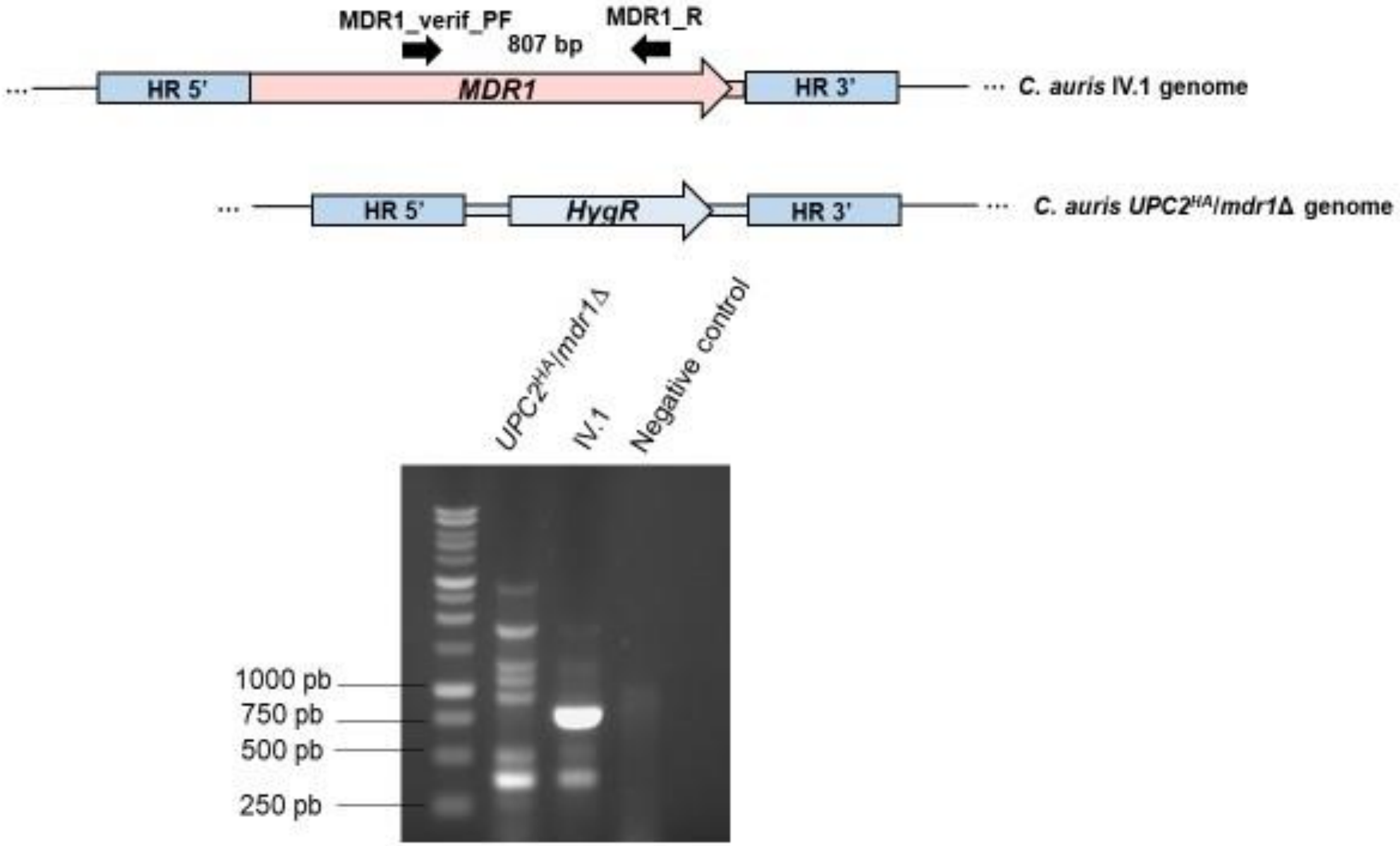

Verification of the deletion of *MDR1* in the *UPC2^{HA}/mdr1Δ* strain. An approximately 800 pb fragment was amplified with two primers located within *MDR1*. As shown on the electrophoresis gel, the PCR product was present in the IV.1 strain and absent in the mutant strain.

Supplementary Figure 8A

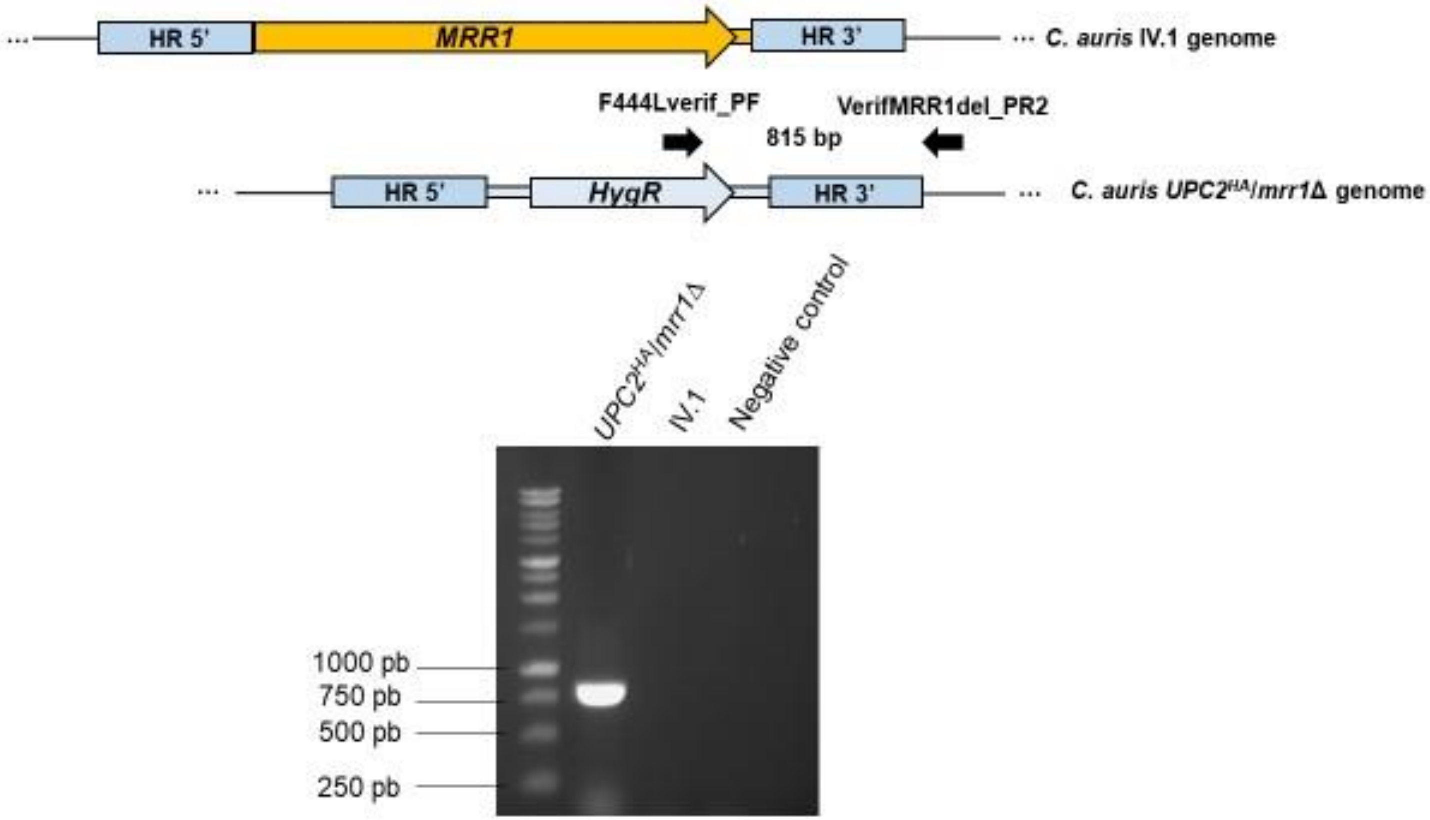

Verification of the deletion of *MRR1* in the *UPC2^HA^/mrr1Δ* strain. An approximately 800 pb fragment was amplified with one primer in *HygR* and another primer in the downstream region of HR 3'. As shown on the electrophoresis gel, the PCR product was present in the mutant strain and absent in the IV.1 strain.

Supplementary Figure 8B

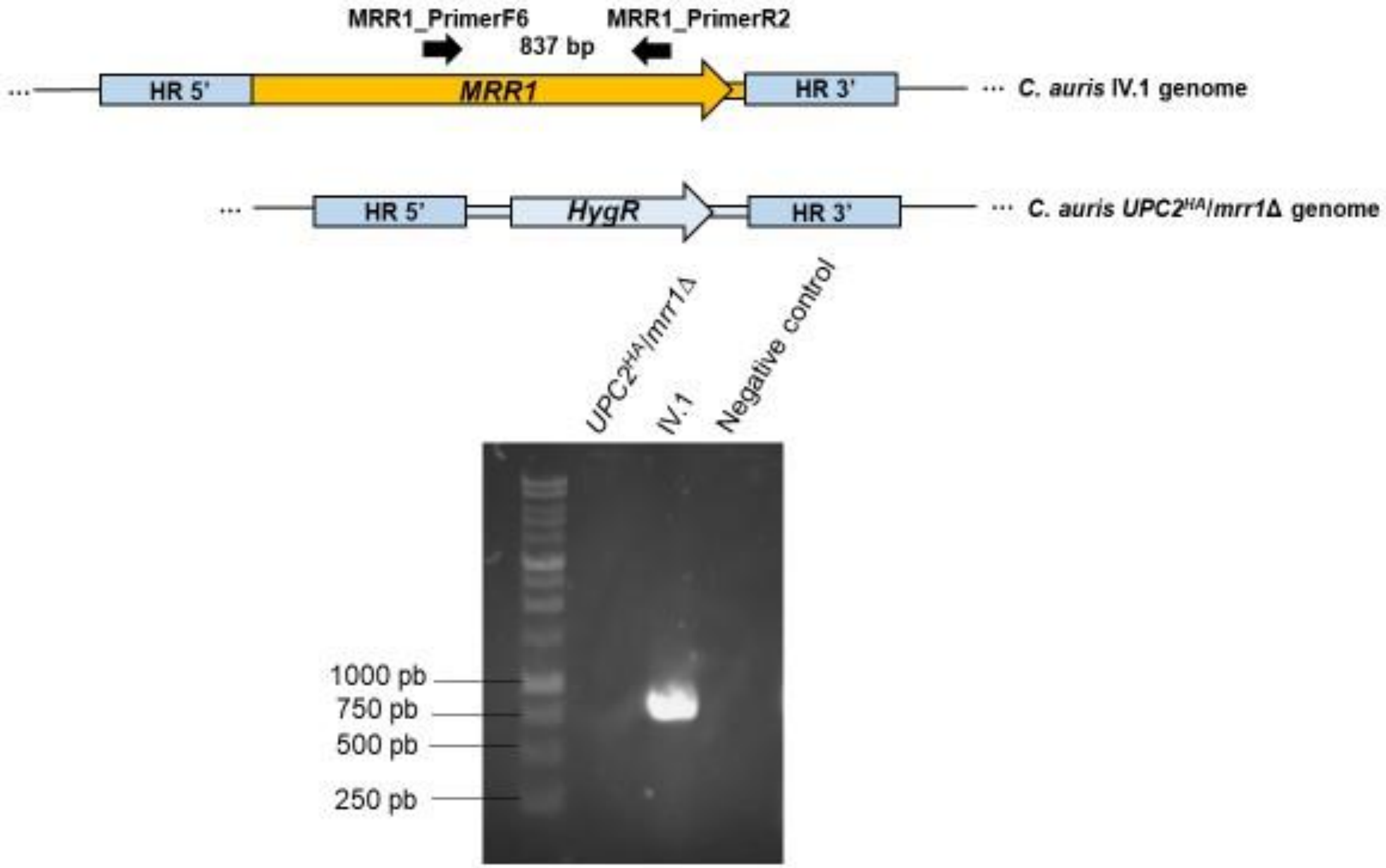

Verification of the deletion of *MRR1* in the *UPC2^HA^/mrr1*Δ strain. An approximately 800 pb fragment was amplified with two primers located within *MRR1*. As shown on the electrophoresis gel, the PCR product was present in the IV.1 strain and absent in the mutant strain.
